# Early functional recovery after two-stage surgery with an allogenic bone graft for baseplate loosening in reverse shoulder arthroplasty: a case report

**DOI:** 10.20407/fmj.2020-002

**Published:** 2020-07-14

**Authors:** Mitsuko Yamada, Shinichi Kato, Kazuaki Mito, Atsuo Furui, Osamu Niwa, Nobuki Terada

**Affiliations:** Department of Orthopaedic Surgery and Restorative Medicine of the Neuro-Musculoskeletal System, Fujita Health University Bantane Hospital, Nagoya, Aichi, Japan

**Keywords:** Reverse shoulder arthroplasty, Baseplate loosening, Two-stage operation, Allogenic bone graft

## Abstract

**Objectives:**

Reverse shoulder arthroplasty (RSA) for cuff tear arthropathy results in good shoulder function. However, RSA is associated with several complications, including infection, dislocation of the shoulder joint, implant loosening, and axillary nerve palsy. Several problems may also occur on the glenoid side, including bone defects of the glenoid, baseplate loosening, and displacement of the sphere. Herein, we report a 79-year-old man who obtained early functional recovery following a two-stage operation with an allogenic bone graft to treat baseplate loosening and a glenoid bone defect after RSA.

**Case report:**

The patient presented with pain during motion and limited active shoulder joint movement 5 weeks after undergoing RSA for cuff tear arthropathy. CT revealed baseplate loosening and a glenoid bone defect; these complications were treated via a two-stage operation. The first stage comprised the removal of all implants and the grafting of allogenic bone from the femoral head into the glenoid defect. Six months later, CT confirmed complete union of the grafted bone and glenoid. The second stage comprised the re-insertion of all implants. Two months after the last operation, the active shoulder range of motion of the affected side was almost identical to that of the contralateral side.

**Conclusion:**

Good early functional recovery was obtained using a two-stage operation for baseplate loosening after RSA. Allogenic bone grafting was effective in the reconstruction of the glenoid defect.

## Introduction

Reverse shoulder arthroplasty (RSA) is an effective treatment for cuff tear arthropathy, as it results in painless improved shoulder function. RSA has been performed for cuff tear arthropathy in older adults in Japan since 2014. However, RSA has several potential complications. Major complications include infection,^1^ dislocation of the shoulder joint and loosening of the implant,^2^ inferior scapular notching,^3^ axillary nerve palsy,^4^ glenoid bone defects,^5,6^ baseplate loosening, and displacement of the sphere.^7^

Herein, we report a case of an older adult man who obtained early functional recovery following a two-stage operation with allogenic bone grafting for baseplate loosening after RSA.

## Case Report

**Figure 1:**
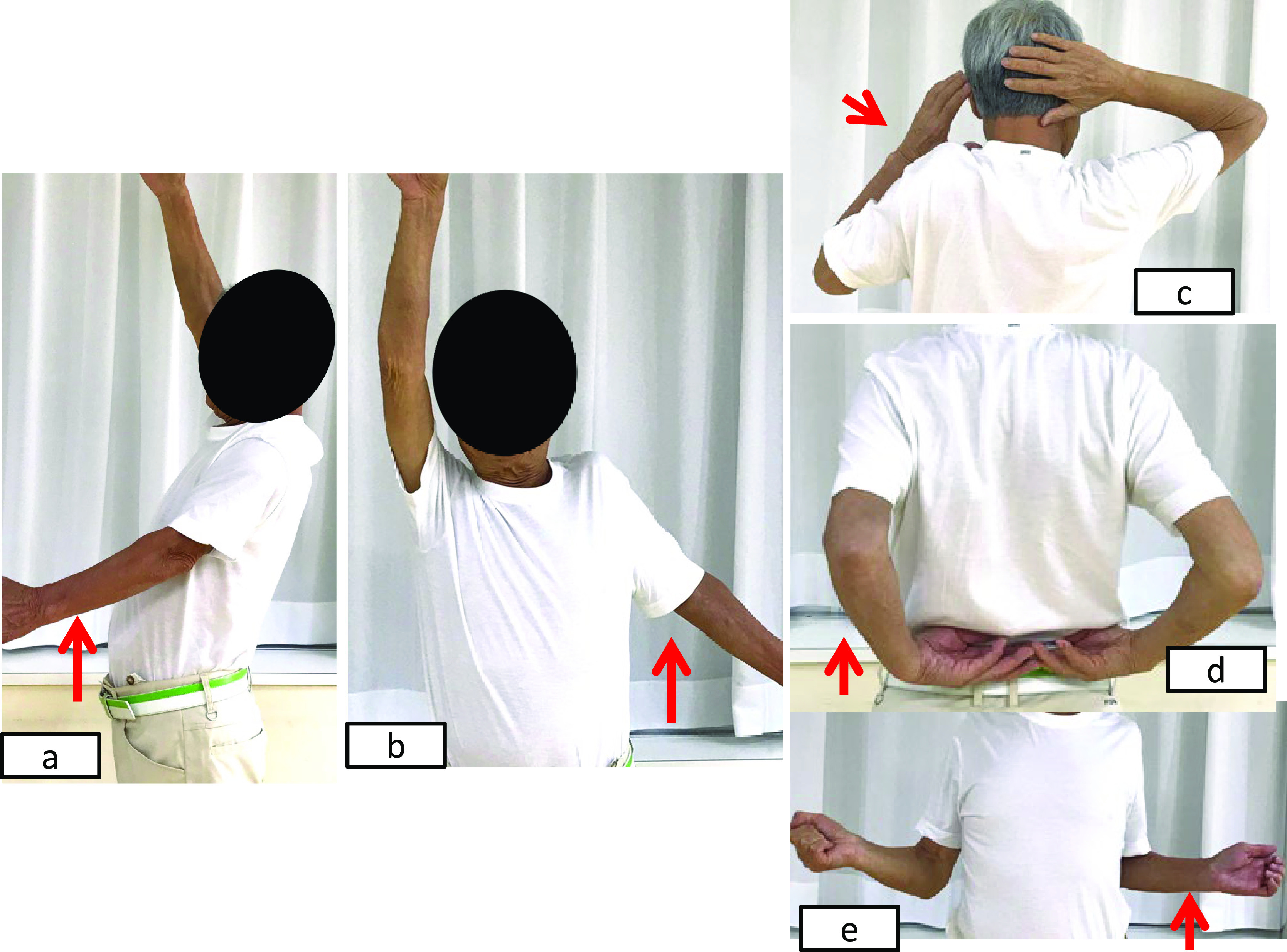
Preoperative left shoulder active movement in a 79-year-old man. a) Elevation of 25°, b) abduction of 45°, c) external rotation of 30°, d) internal rotation to L5.

**Figure 2:**
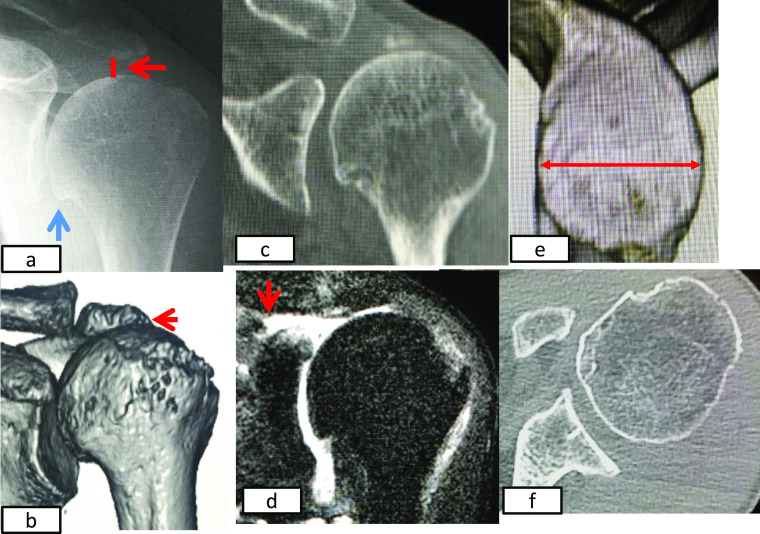
Preoperative images. a) Plain radiography shows an acromio-humeral head interval (AHI) of 2 mm (red arrow) and glenohumeral joint narrowing (blue arrow). b) CT shows a concaved deformity on the undersurface of the acromion. c) Coronal multiplanar view shows that the glenoid shape is type E0 using the Favard classification. d) T2-weighted magnetic resonance imaging shows detachment of the supraspinatus muscle from the greater tubercle to the glenoid rim (red arrow). d) Three-dimensional CT image showing that the transverse length of the glenoid is 25 mm (red arrow), with no glenoid deformity. f) Axial CT image showing that the glenoid shape is type A1 using the Walch classification.

A 79-year-old man presented with limited range of motion (ROM) of his left shoulder. His left shoulder ROM was 40°, 30°, 30°, and to L5 for elevation, abduction, external rotation, and internal rotation, respectively (Figure 1). Plain radiography showed an acromio-humeral interval of 2 mm, with glenohumeral joint narrowing (Figure 2a). Computed tomography (CT) revealed a concaved deformity on the undersurface of the acromion (Figure 2b). Magnetic resonance imaging showed a partial subscapularis tendon tear and global supraspinatus and infraspinatus tendon tears (Figure 2c). Three-dimensional CT imagery revealed that the transverse length of the glenoid was 25 mm, with no glenoid deformity (Figure 2d). The patient was diagnosed with cuff tear arthropathy that was classified as grade 4 using the Hamada classification,^8^ type E0 using the Favard classification,^9^ and type A1 using the Walch classification. The preoperative Japanese Orthopaedic Association (JOA) score was 44.5 out of 80 points due to the poor XP findings and joint stability.

**Figure 3:**
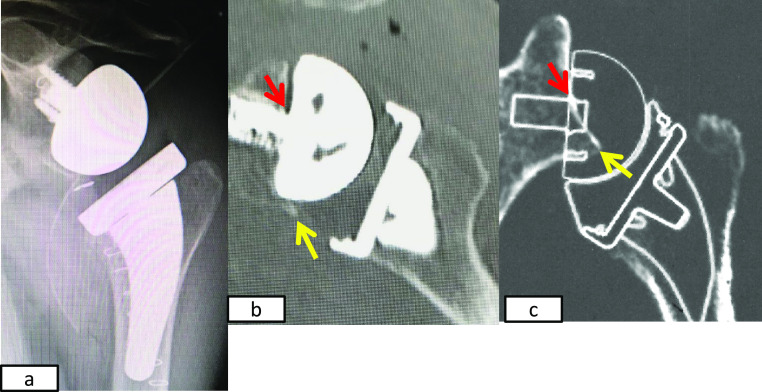
Immediately postoperative images and preoperative planning images. a) Plain radiography. b) Coronal multiplanar image. c) Preoperative planning image. The actual center post of the baseplate (red arrow) is higher than preoperatively planned. The yellow arrow indicates the inferior rim of the glenoid.

In accordance with the JOA guidelines, we decided to perform RSA (Figure 3). The Aequalis Ascend shoulder system (Wright Medical Japan, Tokyo, Japan) was used, with a baseplate size of 25 mm; one compression screw and two locking screws were inserted to fix the baseplate. A lateralized sphere was placed on the baseplate, and the size 3B humeral stem was inserted at 20° retroversion. Finally, the subscapularis tendon was restored. A shoulder brace was used postoperatively.

Postoperative radiography and CT showed that the position of the glenoid component was more superior than preoperatively planned (Figure 3). Three weeks later, the shoulder brace was removed and shoulder ROM exercises were started.

**Figure 4:**
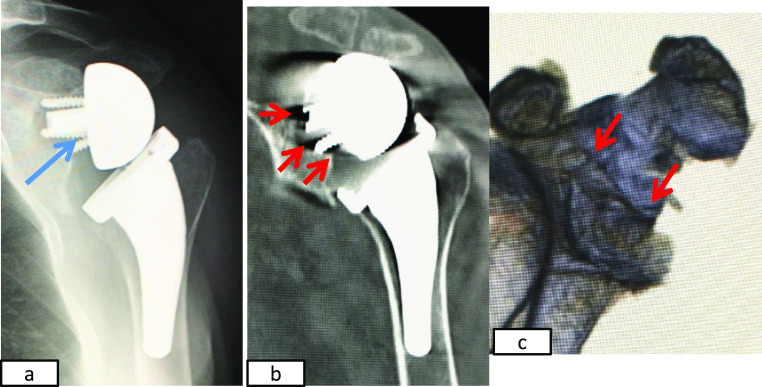
Images from 5 weeks postoperatively. a) Plain radiography shows that the baseplate is displaced superiorly (blue arrow). b) c) Coronal multiplanar and three-dimensional CT images revealing a bone defect at the superior rim of the glenoid (red arrow).

**Figure 5:**
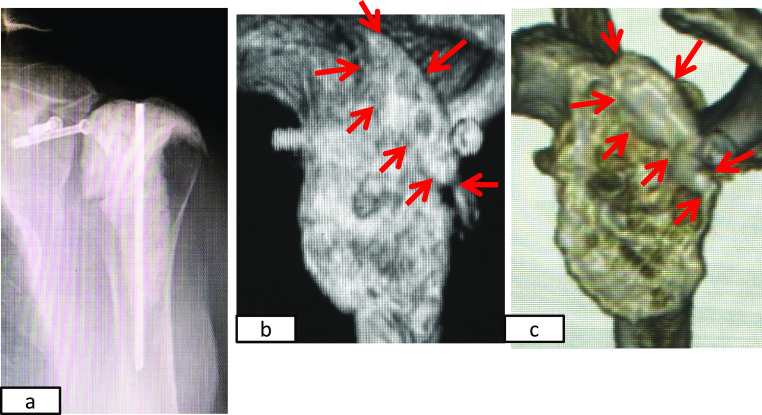
Images obtained after the second operation. a) Radiography showing the two cannulated screws inserted to fix the allogenic bone graft, and a cement spacer inserted in the humeral shaft. b) Three-dimensional CT images taken immediately after the second operation showing the allogenic bone from the femoral head grafted on the bone defect of the glenoid (red arrow). c) Three-dimensional CT images taken 6 months after the second operation showing complete union of the allogenic bone graft (red arrow).

Five weeks postoperatively, the patient reported feeling pain during motion and an inability to actively move the shoulder joint. We found baseplate loosening, and a glenoid bone defect that had been created by the baseplate loosening (Figure 4). This defect had changed the glenoid shape from type E0 to E2 using the Favard classification.^9^ Six weeks postoperatively, we removed all the implants, and grafted allogenic bone from the femoral head into the bone defect with two cannulated screws (3.0 mm diameter) (Meira, Nagoya, Japan). To maintain the length of the humeral bone, a cement spacer shaped like the humeral head was inserted into the humerus (Figure 5).

**Figure 6:**
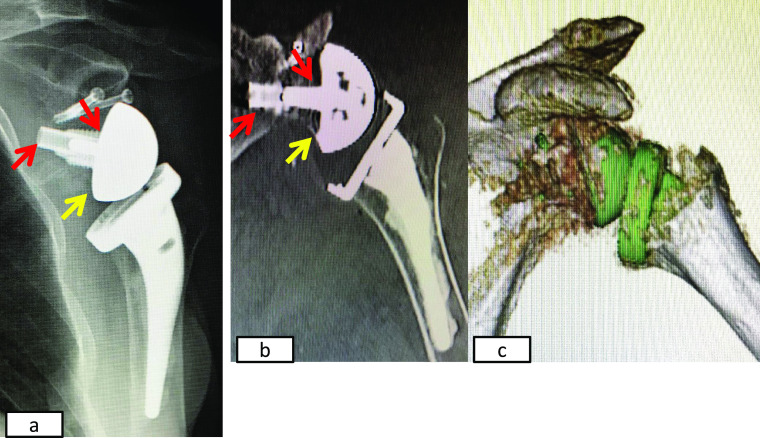
Images obtained 2 months after the final operation. a) Plain radiography. b) Coronal multiplanar CT showing the baseplate with a long post (13 mm) (red arrow) inserted from the inferior glenoid rim (yellow arrow). c) Three-dimensional CT.

**Figure 7:**
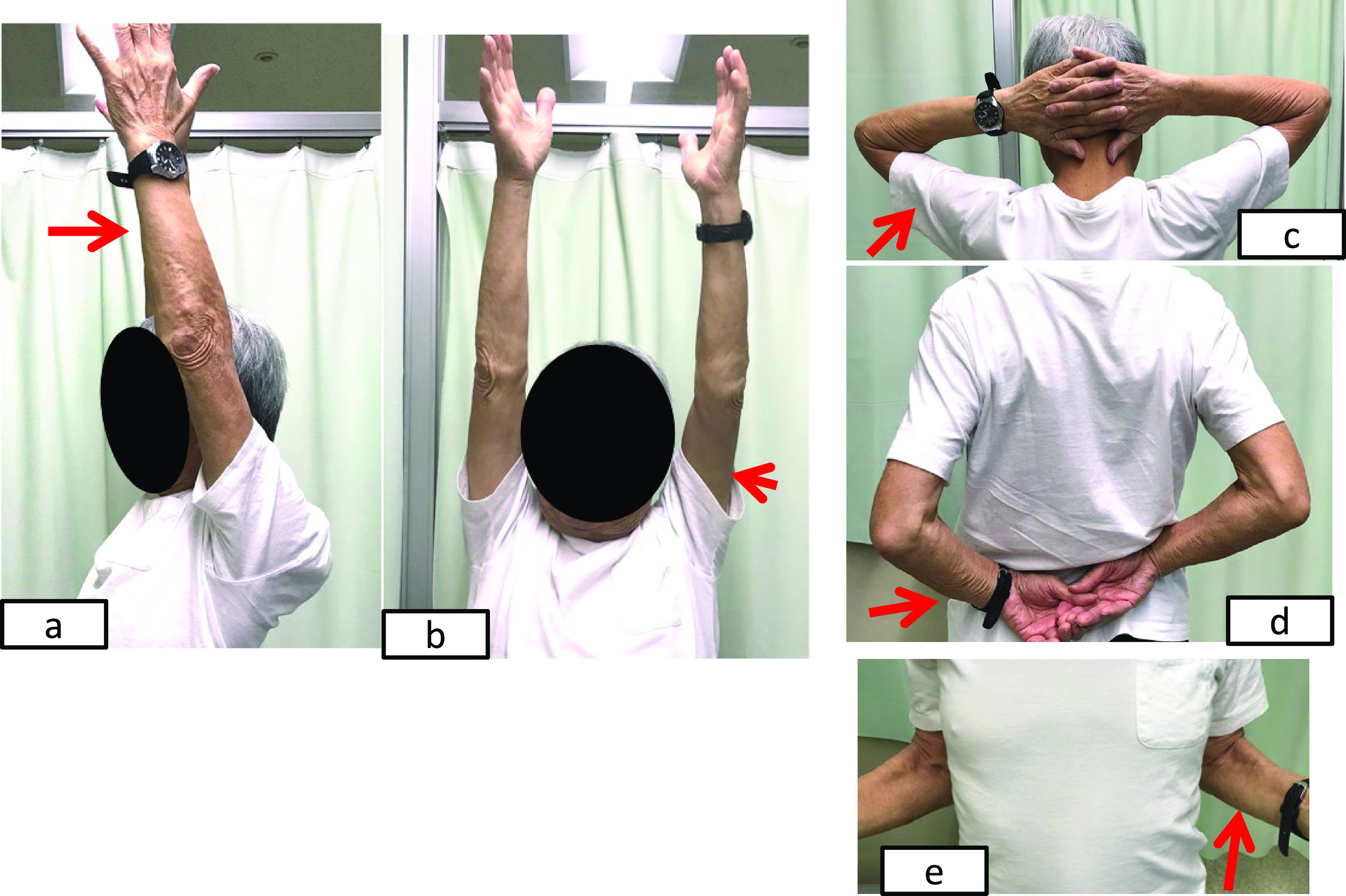
Recovery of range of motion at 2 months after the final operation. The patient achieved the following shoulder active ranges of motion: a) Elevation of 135°. b) Abduction of 175°. c) External rotation of 40°. d) Internal rotation to L5.

Six months after the second operation, CT revealed bone union in the glenoid. We inserted a new glenoid component, a baseplate with a long post (25 mm diameter), one compression screw and two locking screws, and a centered sphere. A size 2B humeral stem was inserted at 20° retroversion with cement (Figure 6). A shoulder brace was used for 3 weeks postoperatively. At 3 weeks postoperatively, a rehabilitation program was started; rehabilitation comprised passive ROM exercise for 2 weeks, and then active assistive and active exercises were added. Two months after the last operation, the left shoulder active ROM was 150°, 150°, 40°, and to L5 for elevation, abduction, external rotation, and internal rotation, respectively (Figure 7). The JOA score was 70.5 out of 80 points.

At the final follow-up performed 1 year after the last operation, the patient had no disability of the left shoulder.

## Discussion

The RSA procedure was invented in 1985.^10^ The goal of RSA is to achieve good active elevation of the shoulder joint without active use of the rotator cuff muscles. The procedure was designed to shift the shaft of the humerus inferolaterally to increase tensioning of the deltoid muscle and move the center of rotation internally to elongate the lever arm of the deltoid muscle. As RSA requires deltoid muscle function for the elevation of the shoulder joint, but does not depend on a functional rotator cuff muscle, shoulder function is maintained even if the rotator cuff muscles are dysfunctional. The main indication for RSA is disability of the rotator cuff muscles, such as cases with a global cuff tear or cuff tear arthropathy.

Many studies have reported good outcomes for RSA; however, a number of complications can occur, including infection,^1^ dislocation of the shoulder joint, loosening of the implant,^2^ inferior scapular notching,^3^ and axillary nerve palsy.^4^ Based on these reported findings, the JOA created strict guidelines for RSA to avoid complications. RSA was introduced in Japan in 2014.

In RSA, complications on the glenoid side include bone defects, displacement of the sphere, and baseplate loosening. One of the reasons for these complications is the glenoid shape. Sirveaux et al. classified glenoid erosion into four types based on anteroposterior radiographs, and showed that the initial radiological appearance of the glenoid affects the inferior scapular notch.^9^ Walch et al. classified glenoid morphology on axial CT imagery, and showed that 40% of cases of primary shoulder osteoarthritis had posterior erosion and dysplasia of the glenoid.^11^

RSA complications also occur due to implant design. A common type of RSA glenoid component is composed of a baseplate that has a central peg and four peripheral screws inserted into the glenoid. Anatomically, the glenoid area is narrow, which makes it difficult to insert four screws in small patients. In addition, there are only two baseplate sizes; therefore, small patients and glenoid components do not match well. In the present patient, the transverse length of the glenoid was 25 mm, which was the same as the diameter of the sphere, and so there was not enough glenoid area in which to insert the baseplate. Therefore, preoperative planning was particularly important to confirm the optimal positioning of the baseplate and other implants.

Another cause of glenoid side complication in RSA is the baseplate position. To avoid glenoid complications, the baseplate must be placed on the inferior part of the glenoid with inferior inclination.^12^ The stability of the baseplate fixation is improved by greater bone density of the glenoid, a longer central peg of the baseplate, and longer screws to provide improved initial glenoid fixation.^13^ In addition, lateralization of the glenoid component effectively increases the deltoid muscle power, but creates more shearing force on the glenoid side, which causes glenoid loosening.

The present patient had early complications after the first operation that manifested as displacement of the glenoid component because of baseplate loosening. We consider that the causes of this complication were that the baseplate was not placed inferiorly on the glenoid as planned, and that a lateralized sphere was used; therefore, we used a centered sphere in the last operation to avoid the creation of a large shearing force.

A previous study compared three options for the treatment of glenoid loosening in RSA: conservative treatment, revision, and hemiarthroplasty.^7^ Conservative treatment and revision reportedly achieved similar outcomes, while hemiarthroplasty achieved the worst outcomes. Furthermore, revision was associated with several complications, and so is only recommended when absolutely necessary.

After the first operation, the present patient had severe shoulder pain during motion on the operated side and had no active range of motion. A bone defect due to baseplate loosening was found in the posterosuperior glenoid, which is an especially important area for the stabilization of the baseplate. To reconstruct the glenoid bone defect, we needed sufficient graft bone and good consolidation of the graft bone and glenoid. The two options for the treatment of glenoid bone defects are autogenic and allogenic bone grafting. Autogenic bone grafts achieve better bone union and lower risk of infection compared with allogenic bone grafts.^14^ We usually use iliac bone as the bone graft at revision surgery; however, for older adult patients, autogenic bone grafting using iliac bone is more invasive than allogenic bone grafting. To achieve sufficient quantity of bone, the iliac bone is not a suitable shape for bone defects of the glenoid. Oze et al. reported good results with RSA using allogenic bone grafts, and concluded that allogenic bone grafts could be used in cases where there was insufficient autogenic bone graft to fill the defect.^15^ Therefore, to obtain an adequate amount of graft bone, we chose to use allogenic bone.

Gupta et al. reported on the management of glenoid bone defects.^6^ They concluded that severe glenoid bone loss can be managed using single-stage bone grafting and RSA. However, a two-stage procedure is recommended when primary baseplate stability is unattainable. Compared with one-stage surgery, two-stage surgery enables clearer confirmation of bone graft consolidation, and better security for the baseplate fixation. Thus, we decided to perform a two-stage operation with an allogenic bone graft.

We needed to wait 6 months before we could confirm bone union in the glenoid. During this period, the patient had undertaken a rehabilitation program to avoid muscle atrophy of the upper limb and contracture of the shoulder joint, including active assistive ROM exercise for the shoulder joint and muscle training below the elbow joint. Therefore, the functional recovery of his shoulder was very rapid after the final operation. Two months after the final operation, his active shoulder ROM was almost identical to that of the contralateral side.

The present case provides evidence that a two-stage operation with an allogenic bone graft is a good option for treating baseplate loosening and a glenoid bone defect after RSA, and achieves good functional outcomes.
